# Transcriptome Analysis Reveals the Negative Effect of 16 T High Static Magnetic Field on Osteoclastogenesis of RAW264.7 Cells

**DOI:** 10.1155/2020/5762932

**Published:** 2020-03-26

**Authors:** Ting Huyan, Hourong Peng, Suna Cai, Qi Li, Dandan Dong, Zhouqi Yang, Peng Shang

**Affiliations:** ^1^Key Laboratory for Space Biosciences and Biotechnology, Institute of Special Environment Biophysics, School of Life Sciences, Northwestern Polytechnical University, Xi'an, China; ^2^Institute of Flexible Electronics (IFE), Northwestern Polytechnical University, 127 Youyi Xilu, Xi'an, 710072 Shaanxi, China; ^3^Research & Development Institute in Shenzhen, Northwestern Polytechnical University, Shenzhen 518057, China

## Abstract

The magnetic field is the most common element in the universe, and high static magnetic field (HiSMF) has been reported to act as an inhibited factor for osteoclasts differentiation. Although many studies have indicated the negative role of HiSMF on osteoclastogenesis of RANKL-induced RAW264.7 cells, the molecular mechanism is still elusive. In this study, the HiSMF-retarded cycle and weakened differentiation of RAW264.7 cells was identified. Through RNA-seq analysis, RANKL-induced RAW264.7 cells under HiSMF were analysed, and a total number of 197 differentially expressed genes (DEGs) were discovered. Gene ontology (GO) enrichment analysis and Kyoto Encyclopedia of Genes and Genomes (KEGG) pathway analysis indicated that regulators of cell cycle and cell division such as Bub1b, Rbl1, Ube2c, Kif11, and Nusap1 were highly expressed, and CtsK, the marker gene of osteoclastogenesis was downregulated in HiSMF group. In addition, pathways related to DNA replication, cell cycle, and metabolic pathways were significantly inhibited in the HiSMF group compared to the Control group. Collectively, this study describes the negative changes occurring throughout osteoclastogenesis under 16 T HiSMF treatment from the morphological and molecular perspectives. Our study provides information that may be utilized in improving magnetotherapy on bone disease.

## 1. Introduction

The static magnetic fields (SMF) are an important element of the earth's mechanical environment. SMF can be clarified into hypo (lower than 5 *μ*T), weak (range from 5 *μ*T to 1 mT), moderate (range from 1 mT to 1 T), and high (stronger than 1 T) SMF according to the magnetic intensity [1–3]. Each organism on Earth usually lives and sustains in 30-60 *μ*T geomagnetic field (GMF). However, man-made SMF can produce High-SMF (HiSMF). For example, Magnetic Resonance Imaging (MRI), one of the most essential medical equipment, can provide 1.5-3 T HiSMF, which is 30,000 to 60,000 times bigger than the natural SMF on earth [4]. To improve the diagnosis and therapy, efforts have been put into increasing the intensity of SMF in medical research areas. Nowogrodzki recently made the world's strongest MRI machines with a 10.5 T magnetic field [5]. Currently, the use of MRI with 7 T in clinical diagnosis has been approved by the Food and Drug Administration (FDA) [6]. At the same time, the high-intensity magnetotherapy, a noninvasion approach, has been applied in the treatment of various diseases including osteoporosis [7], rheumatoid arthritis [8], diabetic wound healing [9], and cancer [10]. Therefore, the application of HiSMF medical equipment is megatrends in the near future. However, the effect of HiSMF on biological objects still needs to be established.

Some pioneering studies revealed the multiple effects of SMF on biological systems, for example, (1) regulate plant functions, growth, and enhance tolerance against environmental stresses [11]; (2) promote chromosome break repair [12]; (3) delay the early development of zebrafish [13]; and (4) accelerate diabetic wound healing [9]. Recently, as a crucial mechanical environment, the effect of SMF on the bone system attracts more attention. Bone is an essential organ of vertebrates, and the remolding process between bone formation and bone resorption needs to keep balanced because the remolding process is sensitive to alterations in the mechanical environment [14]. Turner reported that in SMF, magnetic nanocomposite could stimulate osteoblastic and vasculogenic potentials by mechanically stimulating the progenitor cell function [15]. Osteoclasts play an important role in mediating calcium metabolism and bone resorption. SMF could mediate inhibition of osteoclastogenesis and bone resorption by Receptor Activator of Nuclear Factor-*κ* B Ligand- (RANKL-) induced Akt, GSK3*β*, MAPK, and NF-*κ*B pathways by Kim et al. [16]. Our previous works have revealed the vital role of HiSMF on osteoclasts, such as suppressing human preosteoclasts FLG29.1 cells survival and differentiation [17] as well as inhibiting NF-*κ*-*Β* ligand-induced osteoclastogenesis via mediating the iron metabolism of RAW264.7 cells [4]. We further found that the effect of SMF on osteoclasts differentiation varies with the increase of the magnetic field intensity. Weak and moderate SMF facilitated osteoclastogenesis and bone resorption activity. In contrast, HiSMF (16 T) had a negative effect, which is related with the downregulated expression of osteoclastogenic genes such as matrix metalloproteinase 9 (MMP9), V-ATPase, carbonic anhydrase II (Car2), and RANK. Furthermore, HiSMF altered osteoclast cytoskeleton organization, which destructed the formation of filamentous actin ring and downregulated the expression of integrin *β*3 [18].

To further comprehensively understanding the role of HiSMF on osteoclastogenesis and bone remolding, next generation sequencing (NGS) was used to explore the crucial underlying factor at transcriptome level in this study. By screening and validating key differentially expressed genes between RANKL-induced RAW264.7 cells under HiSMF and normal conditions, the key functional genes profile and related regulatory networks of HiSMF on osteoclastogenesis were drawn to build the foundation of HiSMF-based magnetotherapy on bone metabolic diseases in future. A more profound knowledge of molecular mechanism of HiSMF-induced inhibitory effects on osteoclasts and the relationship between HiSMF and biological responses could render enhancement in the therapeutic method of bone disorders and help to extend novel clinical applications.

## 2. Materials and Methods

### 2.1. HiSMF Exposure System

The superconducting magnet (JASTEC, Kobe, Japan) could create a high static magnetic field (HiSMF) with a corresponding magnetic field intensity of 16 T. In the superconducting magnet, we have established a cell culture platform, which has been described in previous studies [4, 19]. The CO_2_ concentration in the cell culture platform is controlled at 5%, and the temperature is controlled at 37°C. In this work, RAW264.7 cells were placed in 16 T magnetic field.

### 2.2. Cell Culture

The murine osteoclast precursor RAW264.7 cells (the Cell Collection Center of Shanghai, Shanghai, China) were cultivated in *α*-minimum essential medium (*α*-MEM; Gibco, Carlsbad, USA) with 100 units/mL penicillin, 0.1 mg/mL streptomycin, and 10% fetal bovine serum (Gibco, Carlsbad, USA) in 5% CO_2_ atmosphere at 37°C. The cells were induced with RANKL (50 ng/mL, PeproTech, NJ, USA) to differentiate into mature osteoclasts and were continuously exposed to 16 T HiSMF for 3 days. In this study, it considered RANKL-induced RAW264.7 cells treated with the 16 T magnetic field as the HiSMF group and cells cultured in the ground-based condition as the Control group.

### 2.3. TRAP Staining

RAW264.7 cells, at the phase of the logarithmic growth, were seeded into the 96-well plate with a density of 2 × 10^5^ cells per well, and 100 *μ*L of the medium was added to each well. After the cells adhered to the plates, the cell culture medium was changed with osteoclast differentiation induction medium with 50 ng/mL RANKL, and the cells were placed to the HiSMF system for 3 days. TRAP staining assay was applied by using a Leukocyte kit (Sigma-Aldrich, USA) according to the manufacturer's instructions. Briefly, RAW264.7 cells were treated with 4% formaldehyde for 5 min, and 0.1% Triton X-100 was applied for cell permeabilization in 5 min. Then, the cells were stained with the leukocyte acid phosphatase reagent. Finally, the cells were removed from the wells for climbing slides and photographed under a microscope. TRAP-positive cells were defined as cells that have more than three nuclei and were burgundy.

### 2.4. Cell Proliferation Assay

For the cell proliferation assay, RAW264.7 cells were seeded into the 96-well plate with a density of 1 × 10^5^ cells and 100 *μ*L of the medium in each well. After treatment with the HiSMF system, cell proliferation at day 1, day 2, and day 3 were detected by the Cell Counting Kit-8 (CCK8; Beyotime, Shanghai, China) according to the manufacturer's instructions. Briefly, RAW264.7 cells were cultured in the 96-well plate. Then, 20 *μ*L of the CCK8 solution was added to each well (up to 100 *μ*L per well), the cells were continuously incubated at 37°C for 2 hours, and the optical density (OD) values were recorded at 450 nm by a multifunctional microplate reader (Bio-Rad Laboratories, Hercules, CA, USA). The average value of duplicate wells was used as the cell viability value.

### 2.5. Cell Cycle Assay

RAW264.7 cells, at the phase of the logarithmic growth, were seeded in 35 mm dishes with 2 × 10^5^ cells per dish. After 24 hours of culture, the cell culture system was changed with the serum-free medium and cultured for 24 hours for cell cycle synchronization. Immediately after, the medium was changed with induction medium containing 50 ng/mL RANKL, and the cells were placed to the HiSMF system for 3 days. Then, cells were washed twice with ice-cold PBS and fixed with 75% ice-cold ethanol overnight. Subsequently, cells were stained by 50 *μ*g/mL propidium iodide (PI; Sigma-Aldrich, Louis, MO, USA) and 1 mg/mL RNase A (Sigma-Aldrich, Louis, MO, USA) for 60 min. Finally, the cell cycle of samples was performed on a flow cytometer (FACSCalibur, BD, USA).

### 2.6. Library Construction and Sequencing

First, total RNA of RAW264.7 was obtained by using Trizol reagent (Invitrogen, Carlsbad, CA, USA). Second, the mRNA was enriched with magnetic beads and cut into short fragments; the first strand of cDNA was synthesized. After the end repair, base A and the sequencing adapter was added, agarose gel electrophoresis was used to recover the target size fragments, and PCR amplification was performed to complete the entire library preparation. Finally, the constructed library was sequenced using the Illumina HiSeq 2000 by GENE DENOVO (Guangzhou, China).

### 2.7. RNA-Seq Analysis

Low-quality portions of reads and adapter sequences were wiped out by using Trimmomatic (version 0.32) [20]. The amount of paired-end clean reads of each sample was in Table 1. Cleaned reads to the mouse reference genome (version: GRCm38) were aligned by using Bowtie2. Then RSEM program [21, 22] was applied for quantifying the level of gene expression. Differential expression was determined by using the DESeq2 package [23].

### 2.8. Gene Ontology and KEGG Pathway Enrichment Analysis

We used online resources (https://david.ncifcrf.gov) implemented in DAVID to conduct the gene ontology overrepresentation analysis and pathway analysis [24] to associate the identified DEGs with biological functions and processes and pathways. Gene ontology functional annotation consists of three parts, including cellular components (CC), molecular functions (MF), and biological processes (BP). KEGG pathway enrichment analysis was carried out by using the database (https://www.genome.jp/kegg/).

### 2.9. Quantitative Real-Time PCR Analysis

Ten candidate DEGs were selected to confirm the RNA-seq results. Total RNA was isolated from osteoclasts using Trizol (Invitrogen Corp). After the obtained RNA was reversely transcribed and qPCR was performed, the relative expression of mRNA was determined by the CFX96 Touch qPCR system (Bio-Rad Laboratories, Hercules, CA, USA). SYBR Green Real-Time PCR Master Mixes (Applied Biosystems, Carlsbad, CA, USA) were used in this experiment according to the manufacturer's instructions. The primers of candidate DEGs were obtained from the Primer-BLAST online tool [25], and GAPDH was used as the reference gene [4]. The detailed sequences were showed in Table 2. The reaction was performed under the following amplification conditions: initial denaturation at 50°C for 2 minutes, 95°C for 10 minutes, then 40 reaction cycles at 95°C for 15 seconds, and 60 cycles at 60°C. Gene expression was assessed and analyzed by the 2^−*ΔΔ*Ct^ method.

### 2.10. Statistical Analysis

All experiments were biologically replicated 3 times with 3 technical replicates. The results of the experiment were represented by the mean values of three experiments. The data are expressed as mean ± SD. The Student's *t*-test was used to calculate *P* values, and *P* < 0.05 was considered a statistically significant difference. The graphs and statistical analysis in this paper were generated by the GraphPad Prism (version 6, GraphPad Software, California, USA).

## 3. Results

### 3.1. HiSMF Inhibited the Osteoclasts Activity, Cell Proliferation, and Cell Cycle

**Figure 1:**
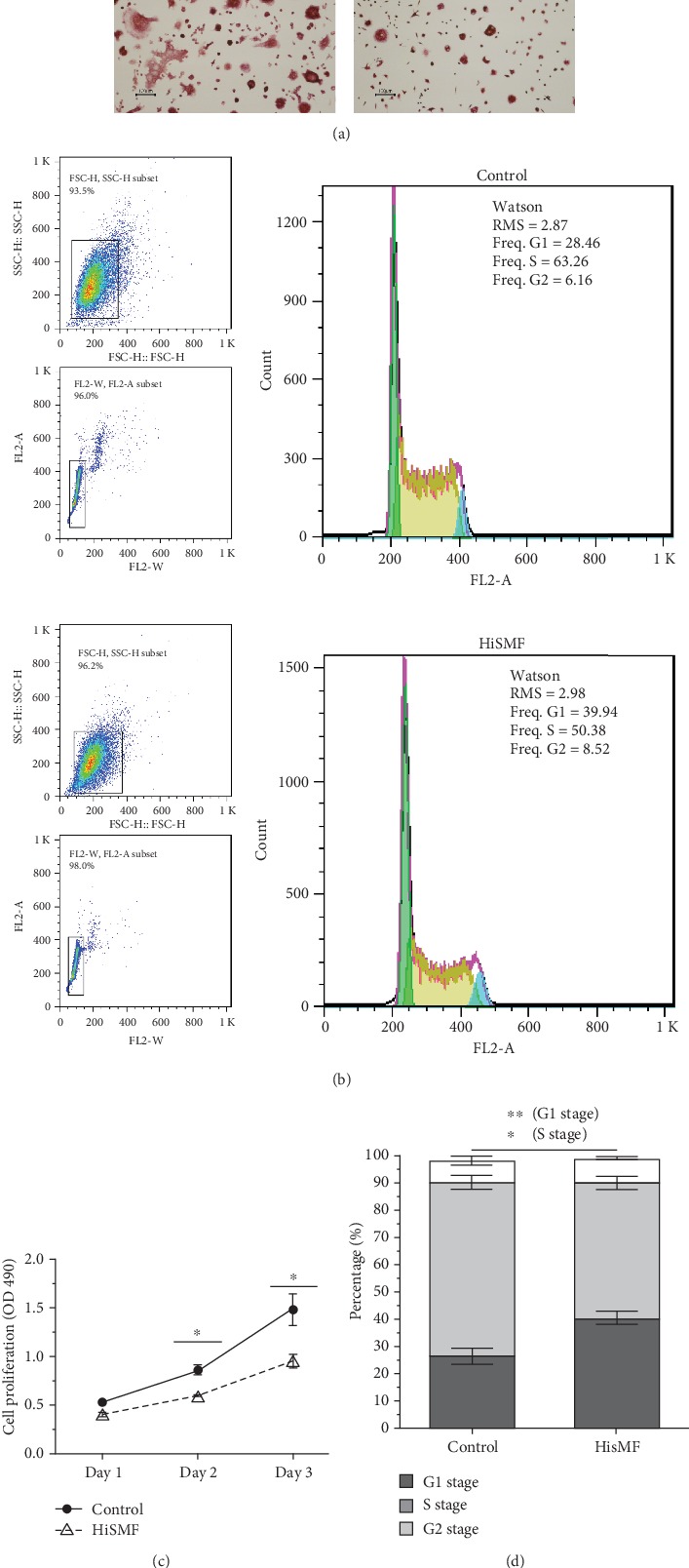
Effect of HiSMF on osteoclastogenesis, cell proliferation, and cell cycle of preosteoclast RAW264.7 cells. (a) TRAP staining of mature osteoclasts generated by RANKL-induced preosteoclast RAW264.7 cells at day 3 (*n* = 3), Bar = 100 *μ*m. (b) Cell cycle distribution of RAW264.7 cells in HiSMF. Cell cycle distribution was determined by flow cytometry with PI staining (*n* = 3). (c) Proliferation of RAW264.7 cells in the Control and HiSMF group. Proliferation of RAW264.7 cells were examined by CCK8 assay, and the results were shown as OD450. (d) The percentage of RAW264.7 cell cycle distribution in the bar graph. All groups were compared with the Control group (*n* = 3). Data shown are mean ± SD, ^∗^*P* < 0.05, ^∗∗^*P* < 0.01.

RAW264.7 cells were inoculated in 18 mm dishes and were induced for 3 days in a HiSMF system with osteoclast induction medium to obtain mature osteoclasts. The TRAP staining results (Figure 1(a)) showed that a larger amount of giant TRAP-positive multinucleated cells were in the Control group than in the HiSMF group. These results indicated that differentiation of RAW264.7 cells was inhibited by HiSMF. To determine whether HiSMF had a negative effect on osteoclastogenesis of RAW264.7 cells, we primarily observed that HiSMF inhibited the proliferation of RAW264.7 cells (Figure 1(c)). For further study, cell cycle distribution was investigated to determine whether it was associated with this inhibitory effect on cell proliferation. It showed that the ratio of cells at the G1 phase raised in the HiSMF group (Figure 1(b)) compared with the Control group. Inversely, the ratio of cells in the S phase had a significant reduction in the HiSMF group versus the Control group. However, the ratio of cells in the G2 phase had no significant difference.

### 3.2. Differentially Expressed Genes (DEGs) Analysis

**Figure 2:**
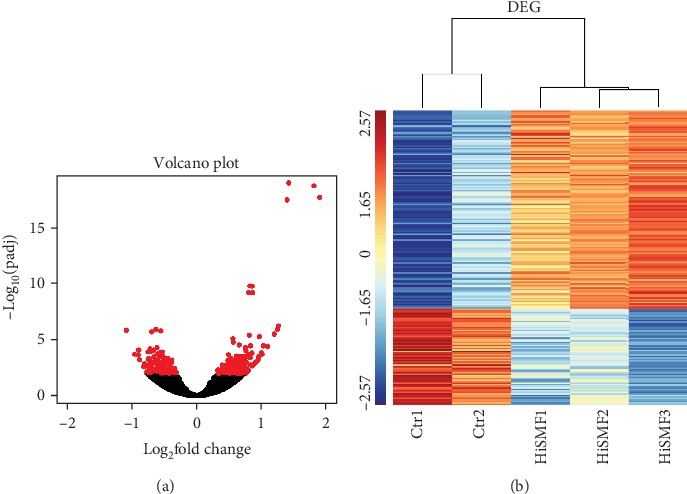
Differentially expressed genes (DEGs) expression profiles of osteoclasts after HiSMF treatment. (a) Volcano plot of DEGs between Control and HiSMF groups. The red dots on the left side of the figure indicate downregulated genes, and the red dots on the right side indicate upregulated genes. (b) Heatmap of cluster analysis of DEGs. Red color indicates highly expressed DEGs, and blue color indicates lower expressed DEGs.

Initially, there were six samples sequenced; however, due to contamination, one of the samples in the Control group was not able to pass the quality control. Therefore, we only used 2 control samples and 3 HiSMF samples sequenced data for further analysis. DEGs were identified between Control and HiSMF groups using the DESeq2 package. The significance level was defined as a false discovery rate (FDR) less than 0.01 and log_2_ fold change (log_2_FC) larger than ±0.5. In total, a number of 197 DEGs were obtained. Upregulated and downregulated DEGs were showed in volcano plots (Figure 2(a)). After identifying the DEGs, we first did a hierarchical cluster analysis and the upregulated and downregulated genes were showed in a heatmap (Figure 2(b)). Gene counts were log_10_ transformed and normalized as *Z*-score. In Figure 2(b), two clusters were clearly displayed; all control groups were in one cluster and the HiSMF groups in another cluster, which showed high intragroup consistency and high intergroup variability.

### 3.3. Enriched GO Ontology and Pathway Analysis of DEGs

**Figure 3:**
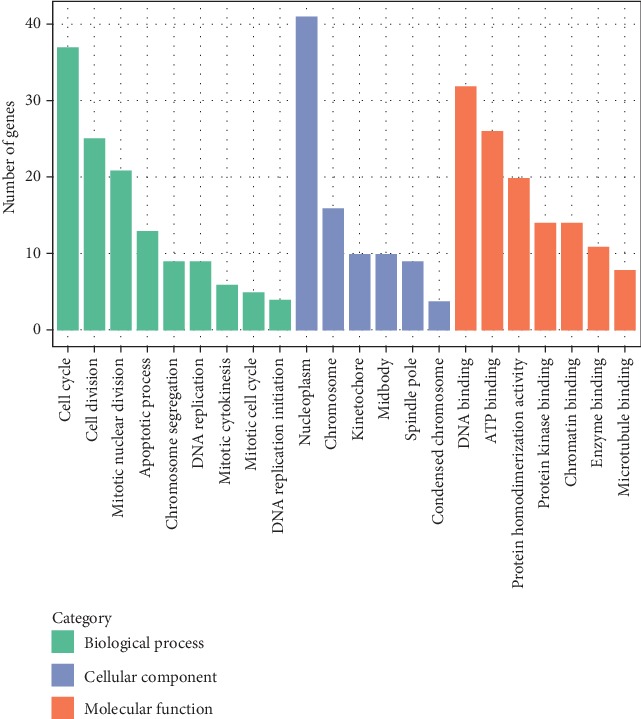
GO enrichment classification map of differentially expressed genes (DEGs). GO enrichment analysis consists of 3 parts: biological processes (BP, green bars), cellular components (CC, blue bars), and molecular functions (MF, orange bars). The *y*-axis is the number of DEGs enriched in each part.

The GO function annotation and Kyoto Encyclopedia of Genes and Genomes (KEGG) pathway provide background knowledge on gene function classification and gene function research. We performed GO analysis and pathway analysis on DEGs, taking *P* < 0.05 as a significance threshold. Based on the GO analysis results, a GO enrichment classification map of DEGs was drawn (Figure 3). From Figure 3, the GO function enrichment results showed that significant biological process (BP) in our analysis were cell cycle, cell division, and mitotic nuclear division. DEGs involved in these processes were Benzimidazoles 1 homolog beta (*Bub1b*), Retinoblastoma-like protein 1 (*Rbl1*), Ubiquitin-conjugating enzyme E2C (*Ube2c*), Kinesin family member 11 (*Kif11*), and Nucleolar spindle associated protein 1 (*Nusap1*). These genes have been considered as the important factors in regulating cell cycle and spindle assembly processes. Therefore, we chose these genes as candidate genes for validation of RNA-seq results. In cellular component (CC), the nucleoplasm had the highest number of genes. In Molecular Function (MF), it was mainly concentrated in DNA binding, ATP binding, and protein homodimerization activity.

**Figure 4:**
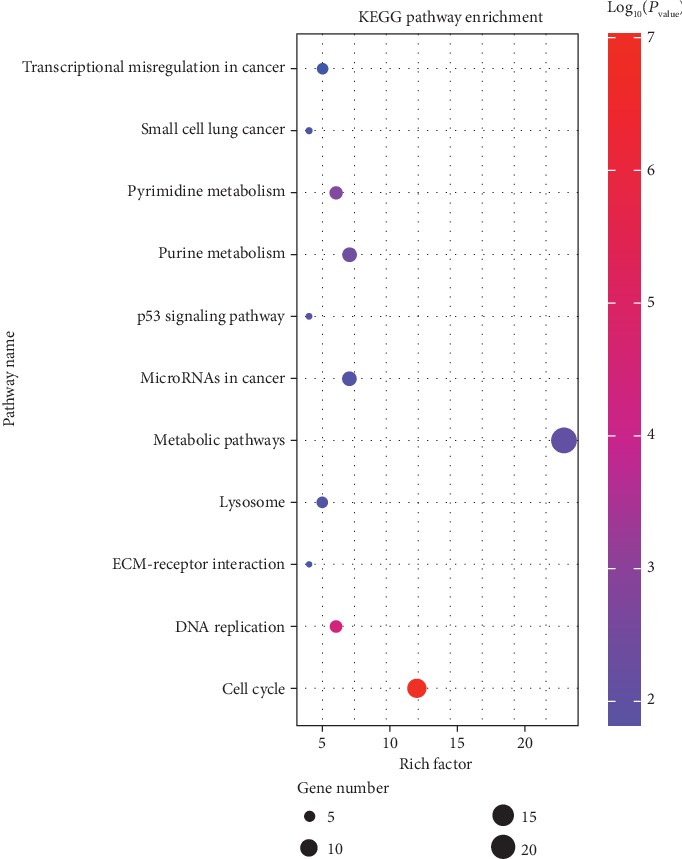
The bubble map of KEGG pathway enrichment analysis of differentially expressed genes (DEGs). The size of the bubbles represents the number of DEGs enriched in each pathway. The color of the bubbles represents the significance level.

In the KEGG enrichment analysis, significant pathways were plotted in the bubble diagram, which shows that DEGs were mainly in the cell cycle and DNA replication-related pathways (Figure 4). Genes involved in these pathways are considered to be candidate DEGs for validation. Therefore, proliferating cell nuclear antigen (*Pcna*, the DNA replication related gene) and cathepsin K (*CtsK*, the marker gene of osteoclastogenesis), which were vital genes in cell proliferating and differentiation, were selected for further confirmation analysis. Besides these well-known genes, there were also some new genes which refer to the sequence known, but its biological function has not been experimentally confirmed. Therefore, we were interested in testing these new DEGs, and then the top 3 new genes in DEGs were also selected as the candidate genes for validation.

### 3.4. Validation of the DEGs

**Figure 5:**
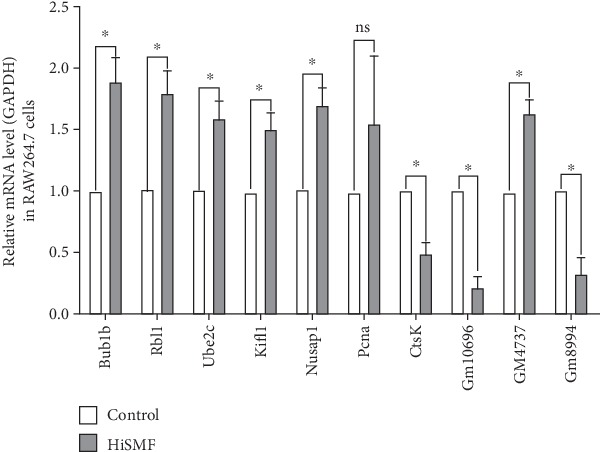
The validation of 10 selected DEGs through qPCR. Relative mRNAs expression level was calculated by fold change. Each group represented as the mean ± SD from three separate experiments (^∗^*P* < 0.05).

Based on DEGs analysis, Go ontology, and pathway analysis, ten candidate genes were selected (Table 3) for further qPCR validation. In these selected 10 DEGs, 7 were upregulated and 3 were downregulated. The qPCR assay was applied on these mRNAs to verify the results of RNA-seq. The results of qPCR confirmed that the expression trends of 9 DEGs coincided to the RNA-seq results except for one gene, indicating that the RNA-seq results were reliable (Figure 5), which provides valuable information for the downstream analysis. The gene not confirmed by qPCR was *Pcna*. We further took a closer look at this gene and found that the expression of this gene showed large variances across three replicates (large SD in the HiSMF group), leading to the failure of confirmation.

## 4. Discussion

The research on the use of HiSMF technology in magnetotherapy in clinics has been increasing due to its useful effects in many diseases, including cancers, edema, inflammation, wounds healing, and bone disorders [7–10, 26]. Due to its safety and noninvasiveness, magnetotherapy has been approved as an instructive novel strategy by the US Food and Drug Administration (FDA) [16]. Especially, HiSMF has been considered as a regulator in bone metabolism, such as bone formation and bone resorption [27]. Bone remodeling, a dynamic equilibrium between degradation by osteoclasts and formation by osteoblasts, maintains the structural integrity of the bone. Accumulated studies indicated that HiSMF enhanced activities of osteoblasts and inhibited differentiation of osteoclasts [18, 28]. However, most studies focused on osteoblast because of its important role in bone formation, and few studies were related to osteoclast, which is responsible for bone resorption. Recently, our previous studies *in vitro* have suggested that HiSMF could suppress the osteoclast formation in preosteoclast FLG29.1 and RAW264.7 cells [4, 17, 18, 28]. Although, there were a few studies that reported the differential expression genes of osteoclasts responding to HiSMF exposure, the systematical gene expression change at the transcriptome level was not clear. Therefore, we used RNA-seq analysis to identify the molecular mechanisms of HiSMF towards osteoclastogenesis.

In this study, a well-characterized cell linage, RAW264.7 cells were used as the osteoclast precursor cell line because the primary osteoclast precursor cells which originated from bone marrow may raise some issues including availability and variation in response pattern for cellular study [29, 30]. In addition to primary osteoclast precursor cells, RAW264.7 cells had been proved to respond to stimuli *in vitro* and differentiate to mature osteoclasts with the hallmark characteristics, so it offered advantages of the cellular model system compared to the primary osteoclast precursor cells [31]. Because of these reasons, we initially performed cytological assays on RANKL-induced RAW264.7 cells to determine whether HiSMF can cause different cellular phenotypes of RAW264.7 cells. Consistent with our previous results [4, 18, 28], the amount of TRAP-positive RAW264.7 cells significantly reduced in HiSMF. Moreover, the RAW264.7 cells proliferation was also decreased in HiSMF associated with the G1 phase arresting. These data confirmed that osteoclastic differentiation and maturation were negatively regulated by HiSMF.

The high throughput NGS has been considered the most comprehensive method for transcriptome analysis and exploring molecular mechanisms [32]. In our transcriptome study, we observed significant changes in the expression levels of 197 genes of RANKL-induced RAW264.7 cells after exposure to HiSMF, of which 133 genes were upregulated, and 64 genes were downregulated (FDR less than 0.01, and log_2_FC larger than ±0.5). Among these DEGs, some of them are new genes. It is important and interesting to identify new genes because the genome of mouse was sequenced, and one of the annotation was predicting genes. As a result, there are many genes predicted by bioinformatics tools, but their functions have not been confirmed by biological experiments. Therefore, these new genes, which the sequence is known, but its biological function has not been experimentally confirmed, were identified to be associated with HiSMF in our study. This provides new information and knowledge of understanding the regulatory network/pathway under HiSMF.

In GO and pathway analysis, the most significant term was cell cycle, indicating that this cellular process was altered in HiSMF. It was consistent with our cytological results. In the GO category of molecular functions, DEGs were mainly involved in DNA binding and ATP binding, suggesting that many factors took part in the transcriptional regulation of RAW264.7 cells. As the primary public pathway-related database, KEGG analysis could effectively enrich signal transduction pathways and metabolic pathways in DEGs [33]. Analogously, in the KEGG pathway analysis, 8 pathways were significantly enriched, including cell cycle, DNA replication, ECM-receptor interaction, lysosome, purine metabolism, metabolic pathways, pyrimidine metabolism, and p53 signaling pathway. Among them, cell cycle was the most significantly pathway and played a vital role in cell growth and differentiation.

Cell cycle has multiple functions in physiological processes, including embryonic morphogenesis, cell proliferation and differentiation, and stem cell pluripotency [34, 35]. It commonly appeared that cell cycle arrest temporally couple with cell differentiation [36]. For the differentiation of osteoclasts, this process is also mediated by cell cycle related genes, such as cyclin-dependent kinase (*Cdk*), Cdk inhibitors, and checkpoint factors [37]. Previous result showed that RANKL could induce osteoclastogenic via arresting cell cycle at the G1 phase in connection with overexpression of the CDK inhibitor p27 [38]. Another work showed that RANKL-induced cell cycle arrest with both upregulation of two Cdk inhibitors (p21 and p27) may be relevant to osteoclastogenesis [39, 40]. In this study, the inhibition effect of HiSMF on cell cycle was coordinated with osteoclast differentiation in cytological and RNA-Seq results.

Through RNA-seq analysis, 10 DEGs involved in cell cycle (*Bub1b*, *Rbl1*, *Ube2c*, *Kif11*, and *Nusap1*), cell proliferation (*Pcna*), cell differentiation (*CtsK*), and newly predicted genes (*Gm10696*, *Gm4737*, and *Gm8994*) were selected for validation by qPCR. Nine out of ten DEGs were confirmed the expression trend. However, one gene, *Pcna*, was not significantly upregulated in qPCR results. It might be due to the inconsistent expression level among replicates, which produces the difficulty of repeating RNA-seq results. In the present study, cell cycle was more systematically analyzed, and the DEGs involved in cell cycle were investigated and validated, which could provide more information for further study.

The five cell cycle related genes in the validated DEGs were all upregulated in the HiSMF group. Among them, Benzimidazoles 1 homolog beta (*Bub1b*), an important spindle checkpoint gene, regulates multiple functional domains, including mitotic timing and mitotic checkpoint control [41]. The upregulation of *Bub1b* may result in chromosomal aneuploidy and instability and finally affect cell cycle process [42, 43]. Ubiquitin-conjugating enzyme E2C (*Ube2c*) could interact with the anaphase-promoting complex/cyclostome (APC/C) to regulate the cell cycle progression [44]. Overexpression of *Ube2c* may participate in the transition of G1 phase to G2 phase [45]. Kinesin family member 11 (*Kif11*), a molecular motor, regulates the separation of centrosome and development of the bipolar mitotic spindle [46]. Upregulated *Kif11* can cause inordinate cell cycle arrest and cell division in mitosis process [47]. Nucleolar spindle associated protein 1 (*Nusap1*), a microtubule-associated molecular, plays an important role in the aggregation of microtubule with mitotic chromosomes during cell cycle regulation [48]. Retinoblastoma-like protein 1 (*Rbl1*), an important controller of entry into cell division [49], has been demonstrated that its phosphorylation status was in the transition from S to M phase, and its dephosphorylation status was in the G1 phase [50]. Future work needs to explore the phosphorylation status of *Rbl1* in osteoclastogenesis.

Besides genes involved in cell cycle, we also identified an osteoclast-specific marker gene, *CtsK*, which was significantly downregulated in the HiSMF group. The previous study has provided evidence for the expression of the osteoclast-specific markers in SMF condition, such as *TRAP* and *CtsK* [4]. Our results supported and confirmed that the *CtsK* might be a critical molecular target in HiSMF. What is more, *CtsK* could be further investigated in this process, which may demonstrate the molecular mechanism of HiSMF toward osteoclast differentiation.

Based on our findings, there are several experiments to do in the future. Initially, the data measured by cytological experiments could apply as an in vivo study, which is the advanced level to verify the molecular functions in osteoclastogenesis under HiSMF. In addition to cell cycle, other pathways such as DNA replication and metabolic have been proved to be related to essential DEGs, but not further studied. How to explore the other DEGs in combination with multiple processes during osteoclastogenesis is the focus of future work. Thirdly, the function of the predicted genes in our results needs to be further validated and may provide more biological information for the mechanism for the osteoclastogenesis under HiSMF. Finally, current studies mainly analyze the dynamic changes of genes in time series based on RNA-seq technology. In future, it is necessary to apply a more comprehensive gene expression map by using advanced technology, such as single-cell sequencing, which reflects more detail in the intracellular network [51, 52]. We firmly believe that single-cell sequencing data will effectively build more reliable and accurate networks for future work.

## 5. Conclusion

Here, we employed NGS to identify, at the transcriptome level, significant DEGs related to cell cycle, cell division, and osteoclasts differentiation in HiSMF condition. The GO and KEGG functional enrichment analyses of the DEGs revealed that the cell cycle, cell division, and DNA replication play a vital role in the regulation of HiSMF on osteoclasts differentiation. The cell cycle was significantly inhibited. Furthermore, nine out of ten DEGs were confirmed the result, and three of them were new genes, which may be related to the differentiation of RAW264.7 cells into mature osteoclasts. Taken together, these findings provided potential new molecular targets for studying the mechanism of the osteoclast differentiation, which may contribute to improving magnetotherapy on bone disease in the future.

## Figures and Tables

**Table 1:** The number of clean reads in the Control and 16 T-HiSMF treated group.

Sample	Number of reads
CONTROL1_R1.clean.fastq	14253190
CONTROL1_R2.clean.fastq	14253190
CONTROL2_R1.clean.fastq	14084434
CONTROL2_R2.clean.fastq	14084434
EXP1_R1.clean.fastq	14394195
EXP1_R2.clean.fastq	14394195
EXP2_R1.clean.fastq	14270708
EXP2_R2.clean.fastq	14270708
EXP3_R1.clean.fastq	13193553
EXP3_R2.clean.fastq	13193553

**Table 2:** Primer sequences of selected 10 target genes used for quantitative real time PCR [4, 25].

Gene name	Primer sequences (5′-3′)
*CtsK (ENSMUST00000015664)*	Forward: TTCTGCTGCTACCCATGGTG
	Reverse: TGCACGTATTGGAAGGCAGT

*Ube2c (ENSMUST00000088248)*	Forward: GTTGCCGCGGTTCGAAAAG
	Reverse: TCAGGGATCTTGGCTGGAGA

*Kif11 (ENSMUST00000012587)*	Forward: GCAGAGCGGAAAGCTAATGC
	Reverse: CAAGGTTGCTGCAGTTGTCC

*Nusap1 (ENSMUST00000068225)*	Forward: GTGACCCCAGTTCCTCCAAG
	Reverse: CACCCAGGTTTCTTCGAGCT

*Bub1b (ENSMUST00000038341)*	Forward: GCCCAGAGAAGACCCCTTTC
	Reverse: CGGTCGGTCTTCCACAGAAA

*Rbl1 (ENSMUST00000029170)*	Forward: AAGCCCTGGATGACTTCACG
	Reverse: AGATCAGGTCCAAGCAGCAC

*Pcna (ENSMUST00000028817)*	Forward: CCTGAAGAAGGTGCTGGAGG
	Reverse: TGTTCCCATTGCCAAGCTCT

*Gm10696 (ENSMUST00000161475)*	Forward: TGCCAAGTGAGCATAGTGGG
	Reverse: TGCTTCAGCTACCAAGTGGG

*Gm4737 (ENSMUST00000059524)*	Forward: TCTGTCAGGCATCCGAGGTA
	Reverse: CCTGGGGCTTGATGTTCACT

*Gm8994 (ENSMUSG00000094973)*	Forward: CACACAGGTCGTTCTCGTCA
	Reverse: CGATGTCCCTGAGAATCCGG

*GAPDH (ENSMUST00000073605)*	Forward: TGCACCACCAACTGCTTAG
	Reverse: GGATGCAGGGATGATGTTC

**Table 3:** Differential expression of selected 10 target genes in control and HiSMF treated group.

Gene name	Description	Con-expression	HiSMF-expression	log2FoldChange	Adjusted *P* value
*CtsK*	Cathepsin K	201359	137884	-0.528987973	0.0000315
*Ube2c*	Ubiquitin-conjugating enzyme E2C	270.5	490.4	0.654515154	0.0000886
*Kif11*	Kinesin family member 11	315.5	542.7	0.603638084	0.000208588
*Nusap1*	Nucleolar and spindle associated protein 1	166	312.3	0.674634071	0.000104789
*Bub1b*	BUB1B mitotic checkpoint serine/threonine kinase	442	846	0.726751612	0.0000085
*Rbl1*	RB transcriptional corepressor like 1	176.5	336.3	0.702550004	0.0000322
*Pcna*	Cell proliferation proliferating cell nuclear antigen	1390.5	2320.3	0.61344962	0.0000102
*Gm10696*	Predicted gene 10696	453.2	216.34	-0.880892972	0.000000216
*Gm4737*	Predicted gene 4737	623.7	1047.2	0.619442963	0.00000731
*Gm8994*	Predicted gene 8994	407.5	216.4	-0.708009712	0.000119099

## Data Availability

The data used to support the findings of this study are available from the corresponding author upon request.
